# Sex Reversal in Zebrafish *fancl* Mutants Is Caused by Tp53-Mediated Germ Cell Apoptosis

**DOI:** 10.1371/journal.pgen.1001034

**Published:** 2010-07-22

**Authors:** Adriana Rodríguez-Marí, Cristian Cañestro, Ruth A. BreMiller, Alexandria Nguyen-Johnson, Kazuhide Asakawa, Koichi Kawakami, John H. Postlethwait

**Affiliations:** 1 Institute of Neuroscience, University of Oregon, Eugene, Oregon, United States of America; 2 Division of Molecular and Developmental Biology, National Institute of Genetics, Mishima, Shizuoka, Japan; 3 Department of Genetics, The Graduate University for Advanced Studies (Sokendai), Mishima, Shizuoka, Japan; University of Pennsylvania School of Medicine, United States of America

## Abstract

The molecular genetic mechanisms of sex determination are not known for most vertebrates, including zebrafish. We identified a mutation in the zebrafish *fancl* gene that causes homozygous mutants to develop as fertile males due to female-to-male sex reversal. Fancl is a member of the Fanconi Anemia/BRCA DNA repair pathway. Experiments showed that zebrafish *fancl* was expressed in developing germ cells in bipotential gonads at the critical time of sexual fate determination. Caspase-3 immunoassays revealed increased germ cell apoptosis in *fancl* mutants that compromised oocyte survival. In the absence of oocytes surviving through meiosis, somatic cells of mutant gonads did not maintain expression of the ovary gene *cyp19a1a* and did not down-regulate expression of the early testis gene *amh*; consequently, gonads masculinized and became testes. Remarkably, results showed that the introduction of a *tp53* (*p53*) mutation into *fancl* mutants rescued the sex-reversal phenotype by reducing germ cell apoptosis and, thus, allowed *fancl* mutants to become fertile females. Our results show that Fancl function is not essential for spermatogonia and oogonia to become sperm or mature oocytes, but instead suggest that Fancl function is involved in the survival of developing oocytes through meiosis. This work reveals that Tp53-mediated germ cell apoptosis induces sex reversal after the mutation of a DNA–repair pathway gene by compromising the survival of oocytes and suggests the existence of an oocyte-derived signal that biases gonad fate towards the female developmental pathway and thereby controls zebrafish sex determination.

## Author Summary

Zebrafish has become an important model for understanding vertebrate development and human disease, yet the genetic mechanisms that regulate gonad fate to determine zebrafish sex remain elusive. In this work, we describe a mutation in the *fancl* gene that causes zebrafish to develop exclusively as male due to female-to-male sex reversal. Fancl is a member of the Fanconi Anemia/BRCA pathway involved in the repair of damaged DNA. We find that the sex-reversal phenotype is caused by an abnormal increase of programmed germ cell death during the critical period for zebrafish sex determination in which oocytes progress through meiosis. This abnormal increase in germ cell death compromises oocyte survival, gonadal somatic cells do not maintain the female gene expression profile, gonads become masculinized to testes, and mutants develop into fertile males. Remarkably, we show that the introduction of a mutated allele of the *tp53* (*p53*) tumor suppressor gene into *fancl* mutants rescues the sex-reversal phenotype by reducing germ cell death. We conclude that Tp53-mediated germ cell death alters gonad fate selection in *fancl* mutants by compromising oocyte survival, possibly by eliminating a hypothesized oocyte-derived signal, which alters sex determination in zebrafish.

## Introduction

The existence of two differentiated sexes is common among animals and yet the mechanisms that determine sex are amazingly diverse. Among vertebrates, for instance, some species use primarily genetic factors and others rely on environmental factors to cause embryonic gonads to become testes or ovaries. Genetic sex determination (GSD) includes monogenic as well as polygenic systems, and in monogenic systems the sex-determining gene is usually found on sex chromosomes that evolved from a pair of autosomes after acquiring a novel sex-determining allele (reviewed in [1]). Mammals have an XX/XY sex chromosome system with males as the heterogametic sex, but birds and many reptiles have a ZZ/ZW sex chromosome system with females as the heterogametic sex. Among fish, both sex chromosome systems have been described [2]–[7]. In environmental sex determination (ESD), factors in the environment, such as temperature, control sexual fate [2]. GSD and ESD have long been thought of as distinct mechanisms, but recent data show regulation by both genetic and environmental factors within a single species [8]. In such species, the integration of genetic and environmental factors ultimately tips the bipotential gonads towards the male or the female fate (reviewed in [9]). For example, in medaka, a teleost fish with an XX/XY sex determination system, high temperatures can sex reverse XX females [10].

Despite the vast diversity of primary sex-determining mechanisms, genes downstream in the sex determination pathway appear to be broadly conserved among vertebrates. It has been suggested that during evolution, different species recruited different downstream genes to be the major sex-determining gene, sometimes relatively recently, and that changes at the top of the sex-determining pathway appear to be better tolerated than changes at the bottom of the pathway because they are less likely to have deleterious effects [11]. In mammals, the Y chromosome gene *SRY* (*Sex determining region Y*) is at the top of the sex determination cascade [12]–[16] and acts as a genetic switch that triggers the bipotential gonad to initiate the male pathway (reviewed in [17]). *SRY* however, does not appear to exist beyond therian mammals [18]. In several groups, including mammals, *Dmrt1* (*doublesex and mab-3 related transcription factor 1*) is a downstream gene in the male sex-determination pathway, but in medaka (*Oryzias latipes*), a duplicated copy of *dmrt1* (called *DMY* or *dmrt1by*) is the major sex-determining gene [19], [20] and recent work has shown that *dmrt1* is required for testis development in chickens [21]. Interestingly, *dmrt1by* is absent in most *Oryzias* species [22], showing that the upstream regulators of sex determination can change rapidly.

Teleost fish show a broad diversity of sex determining mechanisms that range from genetic to environmental, from monogenic to polygenic, and from hermaphroditism to gonochorism (two distinct sexes) [2]. Zebrafish, like many other teleosts, have no obvious heteromorphic sex chromosomes [23]–[25]. Adult zebrafish have two differentiated sexes, but have been described to develop initially as juvenile hermaphrodites because all juveniles develop gonads with immature oocytes regardless of their definitive sex [26]–[28]. Zebrafish juvenile gonads contain immature oocytes that progress through oogenesis in about half of the individuals, which become females, but that degenerate in the other half of the individuals, which become males [26]–[28]. Oocytes begin to degenerate in a window of time (20–30 days post-fertilization (dpf)) that lasts several days and varies among individuals and among rearing conditions [26]–[31]. Because the sex of the zebrafish gonad drives secondary sex characters, gonadal sex determines the definitive sex of the fish. Zebrafish depleted of germ cells develop as infertile males [31]–[34] and it has been shown that the presence of germ cells is essential to maintain female fate in zebrafish [31]. We do not yet know, however, the primary genetic mechanisms that cause some zebrafish to become females and others to become males.

To broaden our knowledge of the genetic mechanisms involved in zebrafish sex determination, we studied a *fancl* zebrafish mutant that develops exclusively as male. Fanconi Anemia complementation group L (Fancl, OMIM 608111), along with 12 other Fanconi Anemia proteins, facilitates cellular responses to a variety of stresses, including signals of DNA damage and apoptosis [35], [36] and belongs to the Fanconi Anemia/BRCA DNA repair pathway. In humans, mutations in any of these Fanconi genes can cause Fanconi Anemia (OMIM 227650), a recessive disease characterized by bone marrow failure, high risk of acute myeloid leukemia, development of squamous cell carcinomas of the head and neck, and developmental abnormalities in many organs including gonads, which causes hypogonadism, impaired gametogenesis, defective meiosis and sterility [37], [38]. Fancl is a member of the Fanconi Anemia core complex with a Plant Homeo Domain (PHD) that mono-ubiquitinates Fancd2 and Fanci [39], [40], which co-localize with BRCA1 and BRCA2 proteins in nuclear foci to stimulate DNA repair. A severe allele of human *FANCL* causes a clinical phenotype that includes hematopoietic and skeletal abnormalities that are similar to, or more severe than, those typically observed in patients suffering from a defect upstream in the Fanconi Anemia pathway (H. Joenje, personal communication). We previously identified the zebrafish ortholog of the human *FANCL* gene [41]. Here we show that *fancl* homozygous mutants develop solely as males and that the absence of *fancl* mutant females is not due to female-specific lethality but to female-to-male sex reversal. Results demonstrated that the sex reversal of *fancl* mutants is not due to the absence of germ cells, but to an abnormal increase of germ cell apoptosis that compromises survival of developing oocytes and masculinizes the gonads. We found that reducing germ cell apoptosis by introducing a Tp53 (*p53* or *tumor protein p53*) mutation rescues the *fancl* sex reversal phenotype, and that many double mutants develop ovaries and become females. These results suggest the model that oocytes normally must progress through meiosis to signal the gonadal soma to maintain female development, and point to Tp53-mediated apoptosis of germ cells as a factor that could be targeted by environmental or genetic signals to modify zebrafish sex determination.

## Results

### A Tol2 Insertion Disrupts *fancl* Structure and Transcription

**Figure 1:**
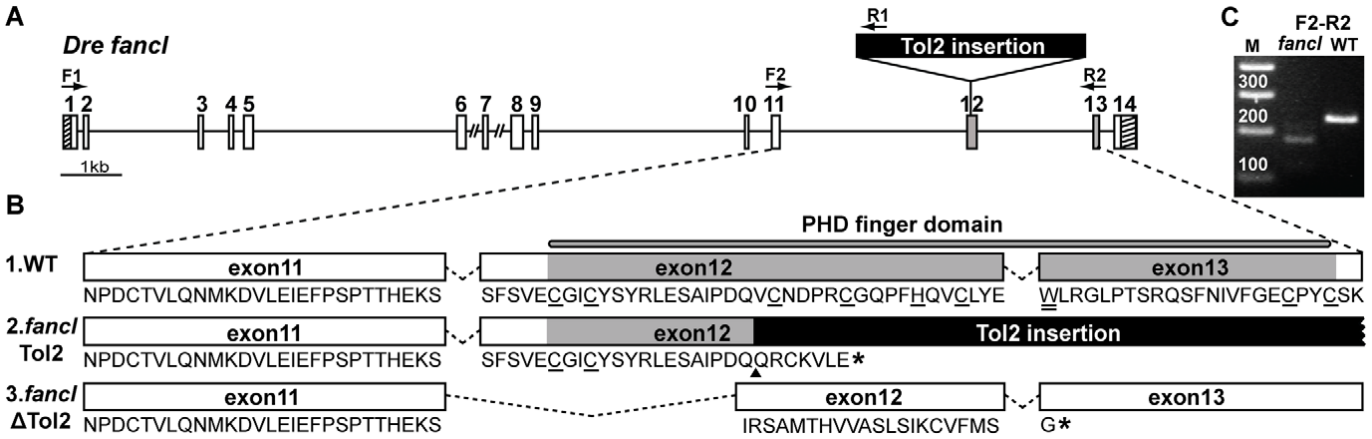
The Tol2 insertion *HG10A* disrupts *fancl* transcripts. (A) Zebrafish *fancl* gene structure showing the Tol2 insertion in exon 12 and the position of primer pairs used for RT-PCR experiments (arrows, F1-R1; F2-R2). Numbered boxes represent exons and dashed boxes indicate untranslated regions. (B) Schematic representation from exon 11 to 13 of the wild-type *fancl* transcript (1.WT) and *fancl* mutant transcripts (2.*fancl*Tol2 and 3.*fancl*D*Tol2*). The PHD finger domain is highlighted in grey. The Tol2 insertion is shown in black and an arrowhead points to its insertion site in the amino acid sequence in B.2. Predicted protein sequences are shown; the highly conserved Cys and His residues are underlined and the critical Trp is double underlined. Asterisks represent premature stop codons. (C) RT-PCR using as template cDNA of adult testes shows that the 232 bp band containing the intact PHD domain in wild types (amplified by F2-R2 primers) is absent from *fancl* mutants. The smaller band (174 bp) amplified in *fancl* mutants corresponds to the *fancl*D*Tol2* transcript in B.3. Abbreviations: M, DNA-Marker.

A zebrafish *fancl* mutant (allele *HG10A*, accession number AB353980) was generated by insertional mutagenesis in a Tol2 transposon-mediated enhancer trap screen [42]. Cloning and sequencing of genomic DNA surrounding the insertion revealed that the Tol2 construct was inserted into exon 12 of *fancl*, thereby disrupting the coding region of the PHD finger domain (Figure 1A and 1B), which is essential for Fancl function [39].

To determine whether the *HG10A* Tol2 insertion disrupts *fancl* transcription, we performed reverse transcriptase-PCR experiments on cDNA isolated from testes of a homozygous *fancl^HG10A^* mutant adult. To learn if the Tol2 insert formed part of the *fancl^HG10A^* transcript, we designed a forward primer in exon1 and a reverse primer in the insertion (F1 and R1 in Figure 1A). The sequence of the PCR product revealed a *fancl^HG10A^* transcript that contained the Tol2 construct inserted after codon Q318 in exon 12 (arrowhead in Figure 1B line 2). This insertion is predicted to insert seven novel amino acid residues and to introduce a premature stop codon (asterisk in Figure 1B line 2), resulting in the loss of 41 of the 57 residues of the PHD finger domain. This loss eliminates the crucial tryptophan-337 (W, double underlined in the wild type (WT) in Figure 1B line 1) that is conserved in all PHD finger-type E3 ligases, as well as histidine-330 and five of the seven cysteines (H and C, underlined in Figure 1B line 1) that are highly conserved in PHD finger domains [39], [43], [44].

To test if *fancl^ HG10A^* mutants could produce *fancl* transcripts with an intact PHD domain due to elimination of the Tol2 insertion, we amplified the region encoding the PHD domain using primers flanking the Tol2 insertion (primers F2 in exon-11 and R2 in exon-13, Figure 1A). RT-PCR experiments revealed that *fancl^ HG10A^* mutants lacked the expected 232 base pair (bp) PCR-product corresponding to the intact PHD domain found in wild-type siblings (WT in Figure 1C), but instead possessed a PCR-product of smaller size (174 bp) (*fancl* in Figure 1C). Cloning and sequencing of the F2-R2 products revealed that the small band from *fancl* mutants was a variant transcript that lacked both the first half of exon 12 and the Tol2 insertion (*fancl*ΔTol2 in Figure 1B line 3). This *fancl*ΔTol2 variant resulted from the joining of exon-11 to the second half of exon-12 due to a splice acceptor site that is newly created at the junction of the Tol2 insertion (Figure 1B line 3). The absence of the first half of exon-12 in the *fancl*ΔTol2 transcript introduced a frameshift that generated an early stop codon (asterisk in Figure 1B line 3) leading to a predicted truncated protein lacking the entire PHD domain. These results show that homozygous *fancl^ HG10A^* mutants have two variant transcripts, both of which encode products lacking an intact PHD finger domain shown to be essential for the ubiquitination function of Fancl [39].

### The Lack of Homozygous *fancl* Mutant Females Is Due to Female-to-Male Sex Reversal

**Figure 2:**
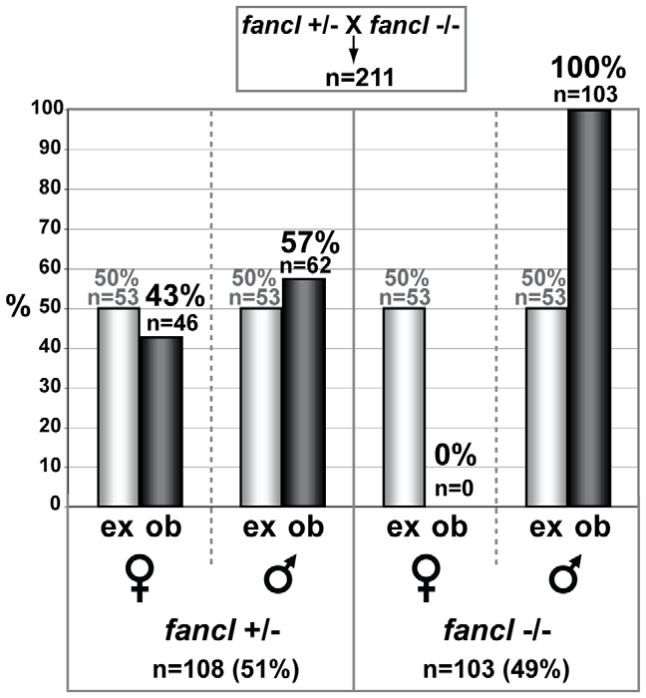
The absence of females in *fancl* homozygous mutants is due to sex reversal. The bar graph represents percentages of expected (ex, grey bars) and observed (ob, black bars) females and males among 211 progeny from a cross of *fancl* heterozygous females (*fancl* ^+/−^) to *fancl* homozygous mutant males (*fancl* ^−/−^). Total numbers (n) and percentages (%) of animals in each category are indicated on the graph. The expected ratio of female heterozygotes to male heterozygotes to female homozygous mutants to male homozygous mutants is 1∶1∶1∶1, but we observed a ratio of about 1∶1∶0∶2 (46 *fancl* ^+/−^ females: 62 *fancl* ^+/−^ males: 0 *fancl* ^−/−^ females: 103 *fancl* ^−/−^ males). This result rules out the hypothesis that homozygous mutant females die, but is predicted by the hypothesis that homozygous mutants that otherwise would have become females develop instead as males.

To characterize the *fancl^ HG10A^* phenotype, we crossed *fancl^ +/HG10A^* heterozygotes (called *fancl* ^+/−^ below), and after genotyping the progeny by PCR, observed that all *fancl^ HG10A/HG10A^* homozygous mutants (called *fancl* ^−/−^ below) developed exclusively as males, even though their wild-type and heterozygous siblings developed about as many females as males. Two alternative hypotheses could explain the lack of homozygous *fancl* mutant females: female-specific lethality or female-to-male sex reversal. To distinguish between these two hypotheses, we crossed female *fancl* ^+/−^ heterozygotes to male *fancl* ^−/−^ homozygotes. We raised 211 progeny to adulthood, determined their phenotypic sex according to sexually dimorphic characters including the color of the anal fin and body shape, and finally scored their *fancl* genotypes by PCR. Under normal conditions, this cross should give 50% heterozygotes (about half of which should be female), and 50% homozygous mutants (about half of which should be female), expecting a 1∶1∶1∶1 ratio of heterozygous females to heterozygous males to homozygous mutant females to homozygous mutant males. The *fancl* female death hypothesis predicts a 1∶1∶0∶1 ratio, or 66% heterozygotes and 33% homozygous mutants, but the sex reversal hypothesis, predicts a 1∶1∶0∶2 ratio, or equal proportions (50%∶50%) of homozygous mutants (all male) and heterozygotes (males plus females). Resulting genotypes revealed 46 *fancl* ^+/−^ females: 62 *fancl* ^+/−^ males: 0 *fancl* ^−/−^ females: 103 *fancl* ^−/−^ males, which showed that about half of the progeny were *fancl* homozygous mutants (103/211, 49%) and the other half were heterozygous for the *fancl* mutation (108/211, 51%) (Figure 2). These results had strong statistical support (chi-square likelihood ratio  = 0.794, p-value >0.1), thus ruling out the hypothesis that homozygous *fancl* mutant females died. Results, however, were consistent with the hypothesis that animals that otherwise would have become females developed as males due to female-to-male sex reversal. Sex distributions within each genotype confirmed our previous observations that all *fancl* homozygous mutants developed as males (n = 103, 100%), and while approximately half of *fancl* heterozygous siblings developed as males (n = 62, 57%), the other half developed as females (n = 46, 43%) (Figure 2). These scores showed strong statistical support for the hypothesis that *fancl* mutants experienced female-to-male sex reversal (chi-square likelihood ratio  = 73.946, p-value<0.0001). To exclude the possibility that some of the *fancl* mutants could have ovaries despite their external male phenotypic characters, we dissected the gonads of adult *fancl* homozygous mutants (n = 45), heterozygous females (n = 11) and heterozygous males (n = 29). In all cases, we found a perfect match between external sexual characters and gonadal sex. These results ruled out the possibility that *fancl* mutants masqueraded as males externally while having female gonads. We conclude that the *HG10A* Tol2 insertion into *fancl* induced a female-to-male sex reversal phenotype in zebrafish.

### *fancl* Is Expressed in Germ Cells of Developing Gonads

**Figure 3:**
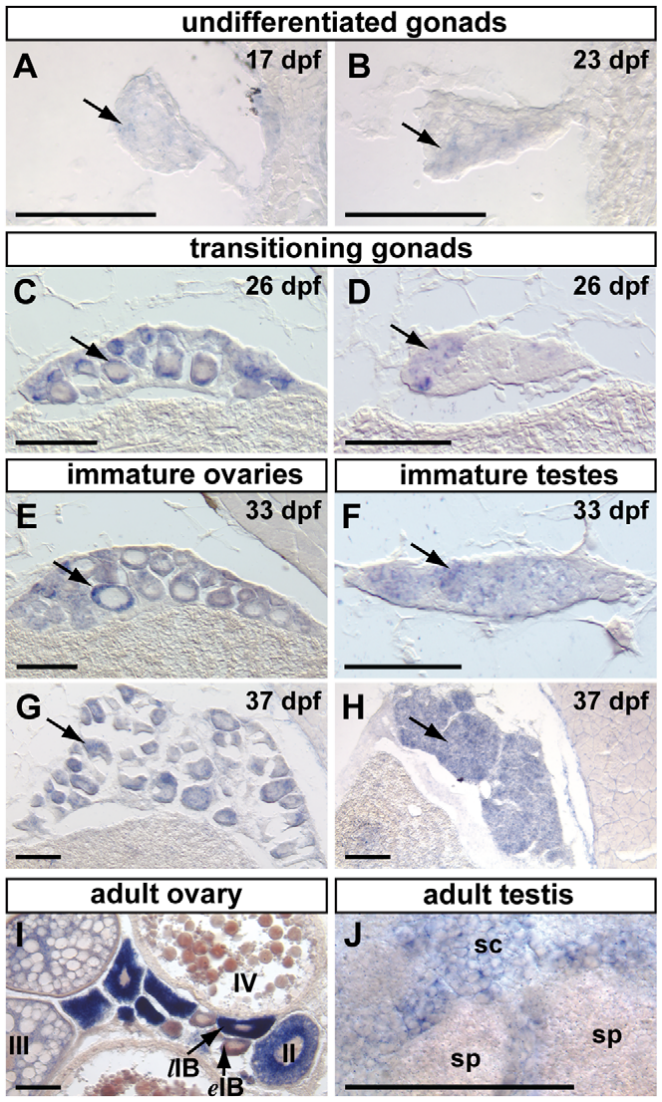
Zebrafish germ cells express *fancl* during gonad development. *In situ* hybridizations with *fancl* probe were performed on cryo-sections of wild-type animals at different stages of gonad development. Weak *fancl* expression signal (arrows) was detected in undifferentiated gonads at 17 days post-fertilization (dpf) (A) and 23 dpf (B). Signal became stronger in germ cells (arrows) of transitioning and immature ovaries (ooplasm of oocytes, arrows in C,E,G) and transitioning and immature testes (D,F,H) at 26, 33, and 37 dpf. In adults, *fancl* expression was restricted to germ cells, but signal intensity depended on the stage of germ cell differentiation. In adult ovaries (I), early stage IB oocytes (*e*IB) already showed low *fancl* expression and late stage IB oocytes (*l*IB) showed strong *fancl* signal in the ooplasm, suggesting that *fancl* expression initiated in early stage IB oocytes. As oogenesis progressed, ooplasm volume increased, cortical alveoli appeared (stage II), yolk accumulated (stage III), and *fancl* expression signal became diluted. In adult testes (J), *fancl* expression signal was detected in a subset of cells with large nuclei and morphology consistent with primary spermatocytes (sc), but signal was not detected in cells with small nuclei in an advanced stage of spermatogenesis (i.e. spermatids and sperm (sp)). Oocyte staging is according to [49] and [29]. Scale bar: 0.1 mm.

Because germ cells play a fundamental role in controlling female sex determination in zebrafish [31], [32], we wondered if *fancl* could play a role in zebrafish germ cell development. To address this question, we first tested whether *fancl* is expressed in germ cells of wild-type zebrafish. We analyzed the expression pattern of *fancl* by *in situ* hybridization on sections of gonads at seven developmental time points encompassing representative stages of gonad development (Figure 3), including sexually undifferentiated and presumptively still bipotential gonads (e.g. 10, 17 and 23 days post-fertilization (dpf)); transitioning gonads (e.g. 26 dpf), sexually determined but still immature gonads (33 and 37 dpf), and mature adult gonads (6 months post-fertilization). Results showed no detectable *fancl* expression in undifferentiated wild-type gonads at 10 dpf (data not shown), but weak expression signal appeared in immature gonads at 17 dpf and 23 dpf (arrows in Figure 3A and 3B). In transitioning gonads at 26 dpf, *fancl* expression increased in developing germ cells (arrows in Figure 3C and 3D), and signal was clearly detected in the ooplasm of oocytes in the ovary-like gonad (arrow in Figure 3C). At 33 dpf and 37 dpf, immature gonads showed a clear morphology of ovaries or testes, and *fancl* expression signal was maintained in developing oocytes and spermatocytes (arrows in Figure 3E–3H).

In adult gonads, *fancl* expression remained restricted to germ cells, but remarkably, the intensity of the detected signal differed depending on the stage of germ cell differentiation (Figure 3I and 3J). In ovaries, the weak *fancl* signal detected in early stage IB oocytes (*e*IB in Figure 3I) contrasted with the obvious strong signal in the ooplasm of late stage IB oocytes (*l*IB in Figure 3I). This result suggests that oocytes up-regulate *fancl* transcription before they transition into stage II. At later stages of oogenesis, *fancl* signal became less intense as oocytes progressed through oogenesis (Figure 3I). This reduction in staining intensity may be due to the dilution of transcript as oocytes increase in volume when cortical alveoli (also known as cortical granules in non-fish species) appeared in the ooplasm (stage II) and yolk began to accumulate (stage III) (Figure 3I). We detected low levels of *fancl* transcript at late stages of oocyte maturation (stage IV), suggesting that *fancl* is part of the maternal load of messenger RNA transcripts stored in the egg and passed along to embryos. This result agrees with our detection of *fancl* transcripts by RT-PCR and in situ hybridization experiments even at early developmental stages before the embryonic transcription machinery becomes active [41]. In testes, *fancl* expression appeared in spermatocytes (sc in Figure 3J), but not in more advanced stages of spermatogenesis, including spermatids and sperm (sp in Figure 3J). This result revealed the stage-specific expression of *fancl* during spermatogenesis.

The finding that *fancl* was expressed in zebrafish germ cells during the time-window critical for gonad differentiation and sex determination (17 to 33 dpf) and was up-regulated in early stages of gametogenesis is consistent with the hypothesis that Fancl plays a specific role in germ cell development and suggests that its disruption might lead to the female-to-male sex reversal phenotype displayed by *fancl* mutants.

### Gonads of *fancl* Mutants Have Germ Cells

**Figure 4:**
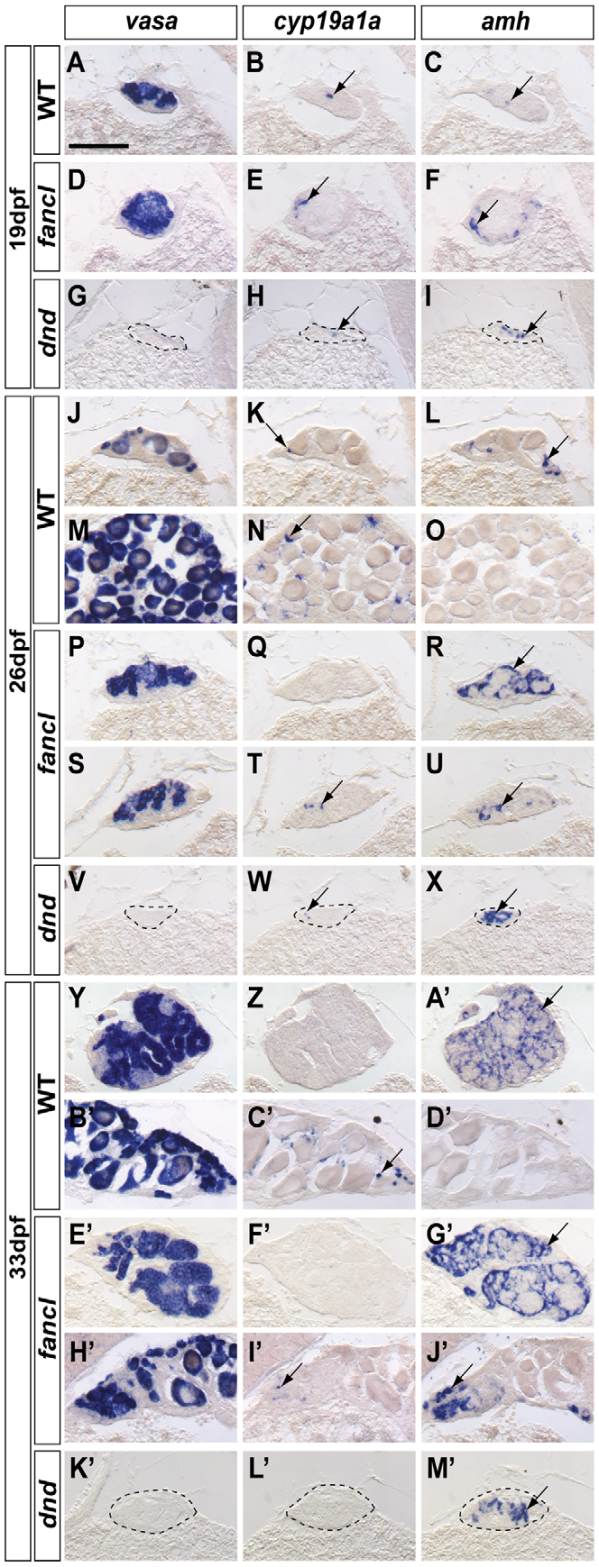
Gonads of *fancl* mutants have germ cells but fail to maintain a female gene expression profile. Comparative expression analysis of the germ cell marker *vasa*, the early female somatic cell marker *cyp19a1a*, and the early male somatic cell marker *amh* in developing gonads of *fancl* homozygous mutants (*fancl*) and their wild-type sibling controls (WT), and in wild-type animals depleted of germ cells by *dead end* morpholino knockdown (*dnd*). To monitor the expression of *vasa*, *cyp19a1a* and *amh*, *in situ* hybridization (ISH) experiments were performed on adjacent cryo-sections of each animal at different stages of gonad development: undifferentiated gonads at 19 dpf (A–I), transitional juvenile gonads at 26 dpf (J–X) and post-transitional juvenile gonads at 33 dpf (Y-M'). Arrows point to examples of regions showing gene expression. ISH with *vasa* probe labeled germ cells in wild types (A,J,M,Y,B') and *fancl* mutants (D,P,S,E',H'), and confirmed the depletion of germ cells in *dnd* animals (G,V,K'). In undifferentiated gonads at 19 dpf, female and male markers were expressed in all genotypes: WT (B,C), *fancl* (E,F) and *dnd* knockdown animals (H,I), showing that the onset of *cyp19a1a* and *amh* expression does not depend on germ cells or on *fancl* function. At 26 dpf, controls had started to enter either the male pathway by down-regulating *cyp19a1a* and up-regulating *amh* (K,L) or conversely into the female pathway by up-regulating *cyp19a1a* and down-regulating *amh* (N,O), correlated with the presence of few or many oocytes, respectively. In contrast, most 26 dpf *fancl* mutants already showed a male expression profile by the absence of *cyp19a1a* and the up-regulation of *amh* (Q,R) and only one *fancl* mutant showed a low number of *cyp19a1a*-expressing cells while nevertheless maintaining high *amh* expression (T,U). Except for *vasa*, expression profiles of 26 dpf *dnd* knockdown gonads were similar to *fancl* mutants (W,X). At 33 dpf, wild-type controls showed either a male expression profile (no *cyp19a1a* and high *amh* expression, Z,A') or a female expression profile (high *cyp19a1a* and no *amh* expression, C',D'). Most 33 dpf *fancl* mutants showed a male expression profile (F',G'), even if gonads maintained an ovary-like morphology (I',J'). All 33 dpf *dnd* animals showed a male expression profile (L',M'). Scale bar: 0.1 mm (A).

Because zebrafish depleted of germ cells by *dead end* (*dnd*) morpholino (MO) knockdown [45], [46] develop exclusively as males [31], [32], and even though adult *fancl* mutants are fertile, we wondered if the female-to-male sex reversal of *fancl* mutants could be related to extremely low numbers of germ cells during stages of sex determination in juvenile mutants, or at least in those that otherwise would have developed as females and had been reversed to males. To answer this question, we performed gene expression analyses comparing gonads of *fancl* homozygous mutants (*fancl*), wild-type sibling controls (WT) and *dnd*-MO knockdown animals (*dnd*) at key stages in sex determination: 19 dpf (Figure 4A–4I), 26 dpf (Figure 4J–4X) and 33 dpf (Figure 4Y–4M'). Expression of the germ cell specific marker *vasa* [47] revealed the presence of germ cells in gonads of all *fancl* mutants sectioned (n = 15) (Figure 4D, 4P, 4S, 4E', and 4H') and sibling controls (n = 13) (Figure 4A, 4J, 4M, 4Y, and 4B'), while all germ-cell depleted animals injected with *dnd*-MO (n = 16) lacked *vasa* signal (Figure 4G, 4V, and 4K'). The presence of substantial numbers of germ cells in all *fancl* mutants tested even at early stages of gonad development rules out the possibility that the near absence of germ cells is the cause of the female-to-male sex reversal in *fancl* mutants.

### *fancl* Mutants Fail to Maintain *cyp19a1a* Expression and Fail to Down-Regulate *amh* Expression

Because all *fancl* mutants developed as males, we wondered if *fancl* mutants embark upon the male pathway from the beginning of gonad development, or whether they follow a normal bipotential pathway of development that later derails exclusively to the male pathway. To address these alternatives, we used the expression of *cyp19a1a* (*cytochrome P450 family 19 subfamily A polypeptide 1a*) and *amh* (*anti-Mullerian hormone*), which are the earliest sex-specific somatic gonadal cell markers known for ovary and testis, respectively, to monitor development before gonads were sexually differentiated at the morphological level [29], [31], [48].

In 19 dpf undifferentiated gonads, somatic cells of *fancl* mutants, as well as those of wild-type controls and *dnd*-MO animals, expressed both the female marker *cyp19a1a* and the male marker *amh* (Figure 4B, 4C, 4E, 4F, 4H, and 4I). This result showed no indication that *fancl* mutant gonads were developing abnormally, which suggests that *fancl* mutant gonads initially embark upon the normal bipotential pathway of development, and later derail into the male pathway. The fact that individual gonads in both *fancl* mutants and WT siblings expressed both *cyp19a1a* and *amh*, as did animals lacking germ cells, suggests that the onset of expression of these somatic cell markers is independent of germ cell derived signals. These results extend to a much earlier age than previously noted (19 dpf versus 35 dpf [31]) the time at which gonads depleted of germ cells express *amh*.

At 26 dpf, different individual WT juveniles showed different degrees of sexual differentiation, suggesting that this age is within the transitional period of sex determination. Some WT animals had gonads with few oocytes, low expression of *cyp19a1a* and up-regulation of *amh* (Figure 4K and 4L), while others had gonads with many developing oocytes, up-regulation of *cyp19a1a* and absence of *amh* signal (Figure 4N and 4O). In contrast to WT sibling controls, at 26 dpf, all *fancl* mutants had gonads with no ooctyes or just a few small oocytes, and most of them (4 out of 5) lacked expression of *cyp19a1a* and showed up-regulation of *amh* (Figure 4Q and 4R). Most juvenile *fancl* mutants at 26 dpf, therefore, had completed the transitional period of sex determination, and had embarked on the male pathway. Only one of the five *fancl* mutants analyzed retained a remnant of a few *cyp19a1*a-expressing cells despite the presence of a considerable number of *amh*-expressing cells (Figure 4T and 4U); this animal was probably still transitioning towards the male pathway. In 26 dpf *dnd-MO* animals, all gonads were depleted of germ cells, and like *fancl* mutants, showed no cells or few cells expressing *cyp19a1a* and many cells up-regulated for the male marker *amh* (Figure 4W and 4X). Therefore, most *fancl* mutants and *dnd*-MO animals tipped the fate of the bipotential gonad towards the male pathway earlier than WT controls.

At 33 dpf, WT juveniles had already passed the transitional period of sex determination. Males had immature testes with no oocytes, no *cyp19a1a*-expressing cells and many cells with high levels of *amh* expression (Figure 4Z and 4A'), and females had immature ovaries, with *cyp19a1a*-positive somatic cells surrounding oocytes but no *amh-*expressing cells (Figure 4C' and 4D'). In contrast to WT sibling controls, at 33 dpf, most *fancl* mutant gonads (6 of 8) showed clear testes morphology, including the absence of *cyp19a1a* expression and up-regulation of *amh* expression (Figure 4F' and 4G'). Interestingly, we found two *fancl* mutants that still had some oocytes; in contrast to WT controls, however, these individuals showed low *cyp19a1a* expression and high *amh* signal (Figure 4I' and 4J'), which would be expected if these two *fancl* mutants were putative females that were in the process of sex-reversing to males. At 33 dpf, all *fancl* (Figure 4G' and 4J') and *dnd-MO* animals (Figure 4M') showed the typical male-specific up-regulation of *amh*. In contrast to 33 dpf *dnd-MO* animals, all of which lacked *cyp19a1a* expression (Figure 4L'), *fancl* mutants that still retained some oocytes showed low levels of *cyp19a1a* expression (Figure 4I'). These results would be expected if the presence of oocytes is essential to maintain *cyp19a1a* expression, and suggested the hypothesis that the female-to-male sex reversal of *fancl* mutants is due to abnormal development of oocytes that leads to a failure of somatic cells of the gonad to maintain *cyp19a1a* expression and to down-regulate *amh* expression.

### Oocytes Fail to Progress through Meiosis in *fancl* Mutants

**Figure 5:**
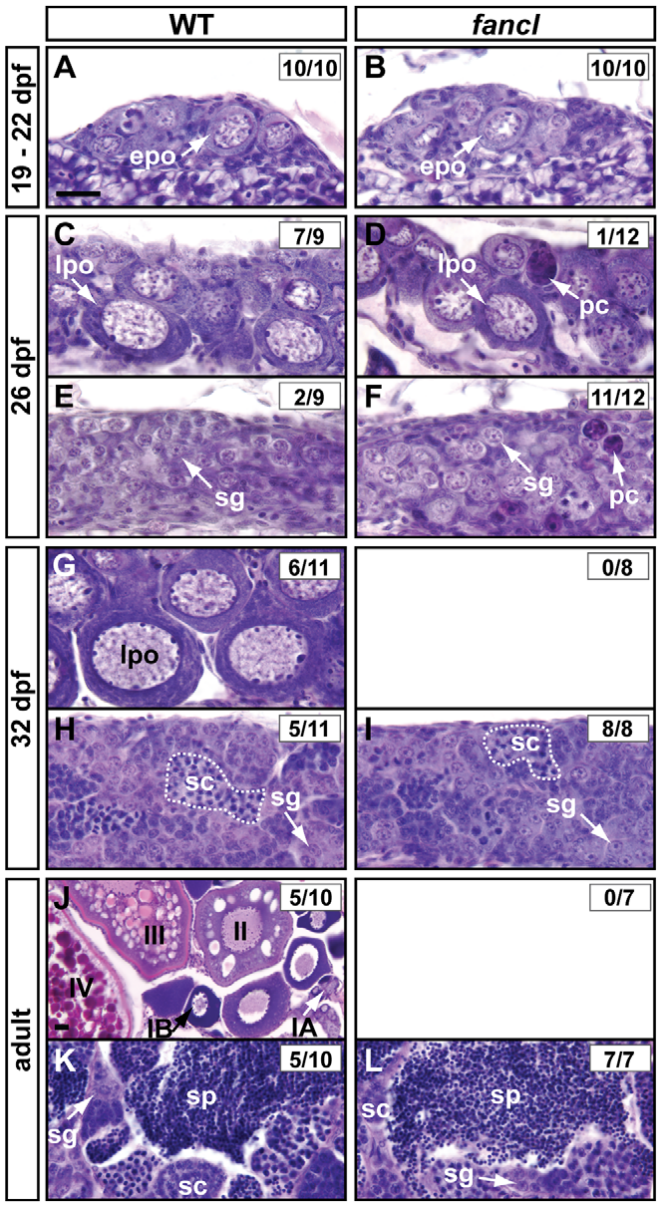
Juvenile gonads of *fancl* mutants contain oocytes that fail to progress through meiosis. Histological comparison of germ cell development in *fancl* homozygous mutants (*fancl*) and wild-type sibling controls (WT), by hematoxylin and eosin staining of gonads at different stages of development: undifferentiated, 19–22 dpf (A,B), transitional juveniles, 26 dpf (C–F), post-transitional juveniles, 32 dpf (G–I) and adults (J–L). At 19–22 dpf, no morphological differences were observed between WT (n = 10) and *fancl* animals (n = 10) and both genotypes showed early stage IB perinucleolar oocytes (*epo* in A,B). At 26 dpf, the first histological differences between WT and *fancl* became apparent. Most WT controls (7 out of 9 individuals) had abundant enlarged perinucleolar oocytes that had progressed from *early* stage IB to *late* stage IB (*lpo* in C), and only two lacked *late* stage IB oocytes (E). In contrast, only one of twelve *fancl* mutant animals had enlarged oocytes at *late* stage IB (*lpo* in D), while the majority (11 out of 12) lacked *late* stage IB oocytes (F). Both wild-type and *fancl* mutant gonads that lacked oocytes possessed spermatogonia (*sg* in E,F). Remarkably, in contrast to wild types, *fancl* mutants showed abundant pyknotic cells (*pc*) at 26 dpf (D,F). At 32 dpf, gonads showed unmistakably the morphology of either ovary or testis, and in wild-type controls, approximately half of the animals (6 out of 11) had ovaries with perinucleolar oocytes at late stage IB (*lpo* in G) and the other half (5 out of 11) showed the typical testis morphology with abundant spermatogonia (*sg*) and spermatocytes (*sc*) arranged in cysts (dashed line in H). In contrast to controls, all *fancl* mutants (n = 8) had gonads that lacked perinucleolar oocytes, and showed testis morphology with groups of spermatogonia (*sg*) and spermatocytes (sc) (I). Finally, in adults, half of the WT controls (5 out of 10) had mature ovaries filled with oocytes at different stages of oogenesis: stage IA, IB, II, III and IV (J), and the other half (5 out of 10) had mature testes (K), in contrast to *fancl* mutants in which all animals (n = 7), had mature testes filled with germ cells at different stages of spermatogenesis: spermatogonia (*sg*), spermatocytes (*sc*) and sperm (*sp*) (L), and none of the seven *fancl* mutants had ovaries. Oocyte stages described according to [49]; Spermatogenesis stages described according to [28]. Scale bar: 0.02 mm (as in A, except for J).

Because the Fanconi Anemia/BRCA system is involved in the repair of damaged DNA, such as that originating in meiotic recombination, we hypothesized that oocyte development is altered in *fancl* mutants. To test this hypothesis, we performed a histological analysis of *fancl* and wild-type gonad sections stained with hematoxylin and eosin at different stages of development to follow the progression of germ cells through meiosis (Figure 5).

At 19–22 dpf, WT sibling controls and *fancl* homozygous mutants had undifferentiated gonads with no obvious morphological differences between genotypes. Gonads of both genotypes contained stage IB perinucleolar oocytes (arrows in Figure 5A and 5B), as indicated by the presence of nucleoli at the periphery of the nuclei [49]. Shortly after the beginning of stage IB, chromosomes decondense and form lampbrush chromosomes [50], which occurs during the diplotene stage of meiosis I as the synaptonemal complex dissolves and recombination nodules keep homologous chromosomes together [51]. We define “*early*” perinucleolar oocytes (*epo*) as stage IB oocytes that have not yet decondensed their chromosomes, and “*late*” perinucleolar oocytes (*lpo*) as stage IB oocytes that have already formed lampbrush chromosomes and entered the diplotene stage of meiosis I. Gonads of *fancl* (10 individuals) and WT siblings (10 individuals) at 19–22 dpf both had *early* (epo in Figure 5A and 5B) but not *late* stage IB oocytes, indicating that at this time, oocytes had not yet entered the diplotene stage of meiosis I in either genotype.

At 26 dpf (Figure 5C–5F), most WT controls (7 of 9 individuals) showed late perinucleolar oocytes that had progressed through meiosis from *early* to *late* stage IB (lpo in Figure 5C), in which lampbrush chromosomes were visible, indicating that recombination had completed and oocytes had already entered the diplotene stage of meiosis I [51]. In contrast to WT, most *fancl* mutants (11 of 12) lacked oocytes at *late* stage IB (Figure 5F), indicating that oocytes in *fancl* mutants failed to progress through meiosis to the diplotene stage. Only one of the twelve *fancl* mutants showed late stage IB oocytes (lpo in Figure 5D), and this individual also contained pyknotic cells (pc in Figure 5D), some of which were identifiable as oocytes and some of which were of unclear origin due to their advanced stage in the process of degeneration. The *fancl* mutants that lacked oocytes (11 of 12) also had numerous pyknotic cells (pc in Figure 5F), and showed groups of spermatogonia (sg in Figure 5F), which were also found in WT animals (sg in Figure 5E) that had gonads with a testis-like morphology.

The difference between *fancl* and WT controls became accentuated at 32 dpf (Figure 5G–5I). At 32 dpf, all *fancl* gonads lacked oocytes and had become immature testes with spermatogonia and spermatocytes (sg and sc in Figure 5I), but only about half of WT siblings had immature ovaries with *late* stage IB oocytes (lpo in Figure 5G) while the other half had immature testes (Figure 5H).

At adult stages (Figure 5J–5L), consistent with results observed at 32 dpf, all *fancl* mutants lacked oocytes and had mature testes filled with germ cells at different stages of spermatogenesis (Figure 5L). In contrast, half of the WT controls had mature ovaries filled with oocytes at different stages of oogenesis (Figure 5J), and the other half had mature testes (Figure 5K).

This analysis of developmental histology revealed that in *fancl* mutants, oocytes failed to progress through meiosis and rarely reached the diplotene stage. Interestingly, in contrast to wild types, we observed abundant pyknotic cells in all *fancl* mutant gonads at 26 dpf (pc in Figure 5D and 5F), suggesting that the absence of oocytes in older *fancl* mutants could be related to increased germ cell apoptosis associated with the failure to complete meiosis.

### *fancl* Mutants Show an Abnormal Increase of Germ Cell Apoptosis

**Figure 6:**
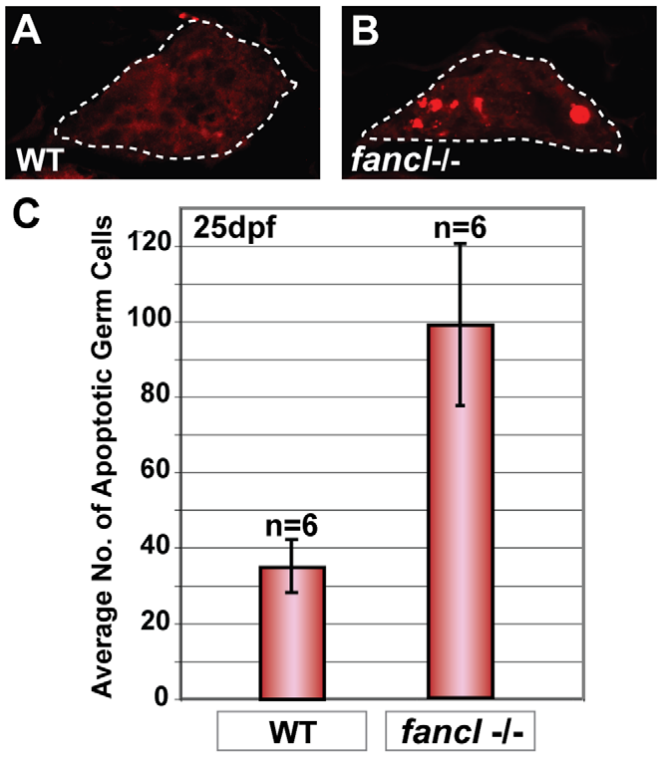
Increased germ cell apoptosis in *fancl* mutants at 25 dpf. Immunodetection of apoptosis by anti-active Caspase-3 in paraffin sections of gonads of wild-type sibling controls (WT) and *fancl* homozygous mutants (*fancl-/-*) at 25 dpf (A,B). Presence of Caspase-3-positive cells (shown in red) was lower in gonads of WT (A) than in *fancl* mutants (B). Gonads outlined by a dashed line (A,B). Bar graph representing the average number of Caspase-3-positive germ cells in each genotype: wild-type sibling controls (WT; n = 6) and *fancl* homozygous mutants (*fancl-/-*; n = 6) at 25 dpf (C). Results showed that the average number of apoptotic germ cells in *fancl* mutants (x– = 99±43) was about three fold higher than in wild-type sibling controls (x– = 35±14), revealing an abnormal increase of germ cell apoptosis in *fancl* mutants at 25 dpf, a critical period for sex determination (C).

To examine whether germ cell apoptosis could be the cause of both the abnormally high number of pyknotic germ cells in *fancl* juvenile gonads and the absence of oocytes at late stage IB, we used immunoassay to examine the activation of Caspase-3, an early marker of apoptosis [52], [53]. We scored the number of Caspase-3-positive cells in 70 gonadal cross-sections in each of 12 individuals: six wild-type sibling controls (Figure 6A) and six *fancl* homozygous mutants (Figure 6B) at 25 dpf, a stage within the time-window critical for sex determination. The morphology of the Caspase-3-positive cells detected in the immunoassay (shown in red in Figure 6B), and the subsequent staining of the same slides with hematoxylin and eosin (data not shown) confirmed that the Caspase-3-positive cells were germ cells and not somatic cells, and corroborated our earlier finding that germ cells that appeared to be pyknotic in our histological analysis are indeed apoptotic cells. In many cases, the shape and size of the apoptotic Caspase-3-positive cells was appropriate for oocytes, however, we cannot rule out the possibility that some Caspase-3-positive cells might be undifferentiated gonial cells (oogonia or spermatogonia). Results revealed that the average number of apoptotic germ cells in gonads of *fancl* ^−/−^ mutants was almost three fold higher than in gonads of wild-type sibling controls (Figure 6C) (t-test p = 0.0058, statistically significant at the p = 0.01 level). Therefore, these results suggest the hypothesis that the absence of oocytes in *fancl* mutants is caused by increased apoptosis of germ cells, especially oocytes, which ultimately leads to the sex reversal phenotype observed in *fancl* mutants.

### Mutation of *tp53* Rescues the *fancl* Female-to-Male Sex Reversal Phenotype by Reducing Germ Cell Apoptosis

**Figure 7:**
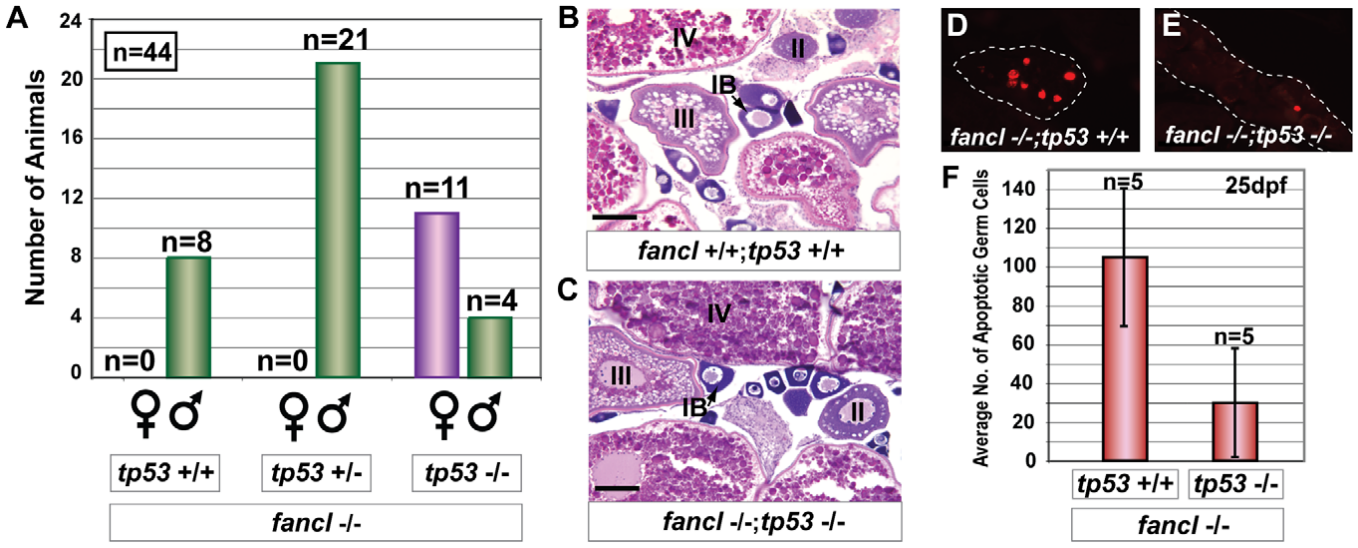
Mutation of *tp53* rescues the female-to-male sex-reversal phenotype of *fancl* mutants by reducing germ cell apoptosis. (A) The distribution of individuals of different *tp53* genotypes among *fancl* ***^−/^*** ^**−**^ homozygous mutant progeny (n = 44) from an in-cross of double heterozygotes (*fancl* **^+/−^** *;tp53* ***^+/^*** ^**−**^) is shown in a bar graph representing the number of females (purple bar) and males (green bars) distributed according to their *tp53* genotypes (wild type, heterozygous or homozygous mutant). Rescue of female-to-male sex reversal was observed exclusively in *fancl* ***^−/^*** ^**−**^ mutant homozygotes that were also homozygous for the *tp53* mutation (n = 15): 11 *fancl* **^−/−^** *;tp53* ***^−/^*** ^**−**^ animals developed as females and 4 developed as males. No rescue was observed in *fancl* mutants that were either wild-type (n = 8; *fancl* **^−/−^** *;tp53* ***^+/^*** ^**+**^) or heterozygous for the *tp53* mutation (n = 21; *fancl* **^−/−^** *;tp53* ***^+/^*** ^**−**^), which all developed as males. Total numbers of animals (n) are indicated on the graph per each sex in each genotype. (B,C) Hematoxylin and eosin staining of gonad sections of wild-type female (*fancl* ^+/+^;*tp53* ^+/+^, B) and rescued female doubly homozygous mutant (*fancl* ^−/−^;*tp53* ^−/−^, C) at adult stage, revealed the presence of morphologically normal ovaries in the rescued *fancl* ^−/−^;*tp53* ^−/−^ females. Ovaries of both genotypes had oocytes at different stages of development (i.e.: IB, II, III, IV). Scale bar: 0.1 mm (B,C). (D,E,F) *tp53* mutation reduces germ cell apoptosis in *fancl* mutants at 25 dpf. Immunodetection of apoptosis by anti-active Caspase-3 in paraffin sections of gonads of *fancl* homozygous mutants simultaneously homozygous wild-type (D) or homozygous mutant for *tp53* (E) at 25 dpf. Dashed lines outline gonad boundaries (D,E). (F) Bar graph representing the average number of Caspase-3-positive germ cells in *fancl^−/−^*;*tp53^+/+^* (n = 5) and *fancl^−/−^*;*tp53^−/−^* (n = 5) at 25 dpf. Results showed that the average number of apoptotic germ cells was approximately three fold lower in doubly homozygous mutant animals (*fancl^−/−^*;*tp53^−/−^;* x–* = *30±56) than their *fancl* ^−/−^ mutant siblings that were wild-type for *tp53* (*fancl^−/−^*;*tp53^+/+^*; x–* = *105±71). This result shows that *tp53* mutation decreased the number of apoptotic germ cells in *fancl* mutants at 25 dpf and demonstrates that the abnormal increase in germ cell apoptosis in *fancl* mutants that compromised the survival of developing oocytes was the mechanism responsible for the female-to-male sex reversal.

The hypothesis that the female-to-male sex reversal of *fancl* mutants is caused by increased germ cell apoptosis predicts that blocking apoptotic pathways should rescue the sex reversal phenotype. Because tumor protein Tp53 (alias p53) is an important activator of apoptosis [54], we can inhibit apoptosis in *fancl* mutants by introducing a *tp53* mutation into the *fancl* mutant line. To generate double mutants, we crossed a zebrafish female carrier of the hypomorphic mutation *tp53* ***^M214K^*** [55] to a male homozygous *fancl* mutant, identified double heterozygotes (*fancl* ***^+/HG10A^***;*tp53* ***^+/M214K^*** called *fancl^+/−^*;*tp53^+/−^* below) among F1 progeny by PCR, and in-crossed double heterozygotes to obtain an F2 population containing double homozygous mutants. Among the F2 raised to adulthood, 44/171 (25.7%), or about a quarter, were *fancl^−/−^* homozygous mutants. Among these 44 *fancl^−/−^* homozygous mutants, 15 were also *tp53^−/−^* homozygous mutants, from which 11 developed as females and four as males (Figure 7A). All of the *fancl* homozygous mutant siblings (n = 29) that were either homozygous wild type for *tp53^+/+^* (n = 8) or heterozygous for the *tp53^+/−^* mutation (n = 21) developed exclusively as males (Figure 7A). This result shows that the female-to-male sex reversal phenotype characteristic of *fancl* mutants was rescued in *fancl^−/−^;tp53^−/−^* doubly homozygous mutants (Figure 7A). The sex-ratio scores observed in the three genotypes showed strong statistical support (chi-square likelihood ratio  = 32.088, p-value<0.0001) for the hypothesis that the presence of females in *fancl^−/−^*;*tp53^−/−^* double mutants and the absence of females in the other *tp53* genotypes (*fancl^−/−^*;*tp53^+/−^* and *fancl^−/−^*;*tp53^+/+^*) is linked to the *tp53* genotype. Histological analyses of *fancl^−/−^;tp53^−/−^* females corroborated the conclusion that external female sex characteristics were accompanied by ovaries filled with normal oocytes at all stages of development similar to *fancl^+/+^*; *tp53^+/+^* wild-type female siblings (Figure 7B and 7C).

To determine whether the *tp53* mutation rescues *fancl* sex reversal phenotype by reducing germ cell apoptosis, we studied the activation of Caspase-3 in histological sections of *fancl* homozygous mutants that were either homozygous for the *tp53^−/−^* mutation (n = 5) or wild-type for the *tp53^+/+^* mutation (n = 5) at 25 dpf, a critical stage for sex determination (Figure 7D and 7F). Counts of Caspase-3-positive cells of 70 gonadal cross-sections per animal in these ten animals showed that double homozygotes (*fancl* ^−/−^;*tp53* ^−/−^) had an average number of apoptotic germ cells approximately three fold lower (Figure 7E and 7F) than their *fancl* ^−/−^ mutant siblings that were homozygous wild-type for the *tp53^+/+^* mutation (Figure 7D and 7F) (t-test p = 0.1032, approaching statistical significance given the small sample size). These results support the hypothesis that the *tp53* mutation rescues the *fancl* female-to-male sex reversal phenotype by decreasing the number of apoptotic germ cells, thereby counteracting the abnormally high frequency of apoptotic germ cells observed in *fancl* homozygous mutants. This result is consistent with the hypothesis that the *fancl* mutation causes the female-to-male sex reversal phenotype by increasing germ cell apoptosis during a critical time for sex determination.

## Discussion

Despite the broad use of zebrafish as a model for vertebrate development, its sex determination mechanism remains poorly understood. In this work, we characterize a zebrafish *fancl* mutation that causes homozygotes to develop exclusively as fertile males due to female-to-male sex reversal. We show that an increase of germ cell apoptosis in mutants compromises the survival of oocytes undergoing meiosis, which may imply an alteration of the signaling between germ cells and somatic cells of the gonads, masculinization of gonads to form testes, and the development of a male phenotype. We show that the mutant sex reversal phenotype can be rescued by reducing Tp53-mediated apoptosis, which allows oocyte survival, and suggests a pivotal role of germ cell apoptosis in zebrafish sex determination. Extending these results from *fancl* mutants to wild-type zebrafish, we propose a model in which genetic and environmental sex determining factors act to increase or decrease germ cell apoptosis and oocyte survival and thus alters the strength of a hypothetical oocyte-derived signal that maintains expression of female genes in somatic cells and hence determines sex in zebrafish.

### Female-to-Male Sex Reversal in Zebrafish *fancl* Mutants Is Due to the Failure of Oocytes to Progress through Meiosis

Fancl protein helps mediate cellular responses to a variety of stresses, especially DNA damage and apoptosis [36]. Mutations in human *FANCL* lead to Fanconi Anemia (FA) [39], a disease of bone marrow failure, enormous risks of cancer, and hypogonadism and impaired fertility (reviewed in [38]). Likewise, the most consistent FA phenotype in murine FA gene knockout models (e.g. Fancc, Fancg, Fanca, Fancd1, Fancd2), is hypogonadism, impaired gametogenesis and infertility (reviewed in [56]). Our work shows that the disruption of *fancl* in zebrafish causes homozygous mutants to develop exclusively as males due to female-to-male sex reversal rather than female-specific lethality. This is the first demonstration, to our knowledge, that a mutation in a Fanconi gene can cause female-to-male sex reversal.

Our work revealed expression of *fancl* in germ cells during zebrafish gonad differentiation, which is consistent with a role of Fancl in germ cell development. Other species, such as mouse, also express *fancl* in their germ cells [57], [58], suggesting a conserved role of Fancl in vertebrate germ cell development. Previous work had shown exclusive male development in zebrafish lacking germ cells due to total loss-of-function of *dead end*, *nanos*, *ziwi*, or *zili* [31]–[34], [59]. We demonstrate here, however, that germ cells are present throughout the entire life in all individuals homozygous for the *fancl* mutation, which rules out the possibility that male development in *fancl* mutants that otherwise would have become females is due to lack of germ cells. Work presented here shows specifically that the mere presence of germ cells is insufficient to feminize gonads, but rather, it suggests that oocytes passing through meiosis are essential to support differentiation of ovaries. Our results are in agreement with previous suggestions that *zili* mutants all become phenotypic males probably due to the lack of oocytes at week 4 during the window of sex determination rather than due to the total loss of germ cells at week 8 [59]. Homozygous *fancl* mutants, in which germ cells are always present, provide a useful tool to better understand the role of germ cell-soma signaling that tips gonad fate towards the male pathway.

Comparison of sex-specific gonadal markers among *fancl* mutants, WT controls and *dnd*-MO animals, which lack germ cells, reveals that the onset of expression of the female marker *cyp19a1a* and the early male marker *amh* in individual undifferentiated gonads at 19 dpf is similar in all genotypes. This result supports the conclusion that the onset of early somatic makers is independent of germ cell signaling [31]. These results also show that undifferentiated gonads of *fancl* mutants initially develop as normal bipotential “juvenile ovaries” containing oocytes at early stage IB with no obvious histological differences from gonads of WT controls.

During the critical time-window for sex determination in zebrafish (e.g. 26 dpf), however, *fancl* mutant gonads become morphologically different from wild-type gonads. Wild-type animals have perinucleolar oocytes that progress through meiosis from early stage IB to late stage IB with obvious lampbrush chromosomes, indicating that recombination is complete and oocytes are at the diplotene stage of meiosis I, in which homologous chromosomes begin to separate but remain attached at chiasmata [51]. In contrast to wild types, most *fancl* mutants lack *late* stage IB oocytes, indicating that oocytes fail to progress beyond pachytene stage, when recombination occurs, and do not enter diplotene. Our results show that the levels of *fancl* transcripts are regulated during the process of gametogenesis because *fancl* expression up-regulates in oocytes transitioning from early to late stage IB (Figure 3I). Consistent with this result, a large-scale gene expression profiling study of developing ovaries in trout found *fancl* in a group of many genes that were over-expressed when the first oocyte meioses were observed [60]. In *fancl* zebrafish mutants, the failure of oocytes to transition from *early* to *late* stage IB suggests that Fancl might promote the successful progression of oocytes through meiosis I or the survival of meiotic oocytes. The FA pathway is apparently involved in meiosis because in mouse, *Fanca* is expressed in pachytene spermatocytes and *Fanca* knockout mice have elevated rates of mis-paired meiotic chromosomes and increased germ cell apoptosis [37]. Whether this effect on meiosis depends on the known role of FA proteins in homologous recombination in somatic cells [61] or some other aspect of meiosis is as yet unknown.

The failure of oocytes to progress through meiosis in *fancl* mutants correlates with the observation that most mutant gonads do not express the female somatic marker *cyp19a1a*, but instead up-regulate the male somatic marker *amh*. Interestingly, we found a few *fancl* mutants with some late stage IB oocytes accompanied by expression of *cyp19a1a*, but also showing high expression levels of *amh*; we interpret these animals as females whose progress towards ovary development was being derailed due to the mutation of *fancl*. These results would be expected if oocytes are essential to maintain *cyp19a1a* expression.

We hypothesize that in juvenile *fancl* mutants, the absence of oocytes progressing through meiosis alters oocyte signaling to the soma that maintains the female program. Without this signal, somatic cells do not maintain the expression of *cyp19a1a*, do not suppress *amh* expression, and as a result, gonads do not become ovaries but instead become masculinized and form testes. It is likely that this signal arising from meiotic oocytes is essential for somatic pre-granulosa *cyp19a1a*-expressing cells to proliferate and to differentiate as mature granulosa cells. In mammals, it has been suggested that meiotic oocytes reinforce ovarian fate by antagonizing the testis pathway [62], [63]. Studies on gonadal somatic cell lineages in mice and medaka, have shown that granulosa cells of the ovary and Sertoli cells of the testis develop from a common precursor [64]–[66]. It is possible that mammalian meiotic oocytes reinforce the ovarian pathway by preventing granulosa cells from trans-differentiating into Sertoli-like cells, because the loss of oocytes in mammals induces maturing follicular cells (or pre-granulosa cells) to acquire Sertoli-like cells characteristics [67]. We hypothesize that the action of meiotic oocytes in preventing pre-granulosa cells from trans-differentiating into Sertoli-like cells is an ancestral function that has been conserved in mammals and fishes. Although our experiments do not address the question of whether somatic cells trans-differentiate in *fancl* mutant gonads, our results are consistent with the hypothesis that *fancl* mutants, which lack oocytes at the diplotene stage of meiosis, can not prevent the trans-differentiation of pre-granulosa *cyp19a1a*-expressing cells into Sertoli-like *amh*-expressing cells. This hypothesized mechanism could explain the disappearance of *cyp19a1a*-expressing cells and the maintenance and proliferation of *amh*-expressing cells in *fancl* mutant gonads that results in gonad masculinization. Future transcription profiling analyses comparing wild-type animals and *fancl* mutants lacking oocytes will help to identify genes involved in oocyte-soma signaling essential for ovary development.

### Increased Apoptosis in *fancl* Mutants Compromises Oocyte Survival and Causes Female-to-Male Sex Reversal

We observed that the loss of oocytes in *fancl* mutants during the time-window of sex determination (25 dpf) is accompanied by an abnormal increase of Caspase-3-mediated apoptosis of germ cells compared to wild-type siblings. This result suggests the hypothesis that the disappearance of meiotic oocytes in *fancl* mutants is due to an increase in germ cell apoptosis, which provides a cellular mechanism for the female-to-male sex reversal phenotype of *fancl* mutants. To test this hypothesis, we suppressed cell death in *fancl* mutants by making them homozygous for a *tp53* mutation. We show that the reduction of apoptosis in *fancl^−/−^;tp53* ^−/−^ double mutants is sufficient to promote the survival of developing oocytes and to rescue the female-to-male sex reversal phenotype of *fancl* mutants. Our result showing that only *fancl^−/−^;tp53* ^−/−^ double mutants developed any females, while their *fancl^−/−^;tp53* ^+/−^ and *fancl^−/−^;tp53* ^+/+^ sibling controls developed exclusively as males, indicates that the amount of germ cell apoptosis alters sex determination in *fancl* mutants.

The double mutant experiments further show that Tp53 activity mediates increased apoptosis associated with the *fancl* mutation. Doubly homozygous *fancl^−/−^;tp53^−/−^* rescued females were fertile and developed normal ovaries full of oocytes maturing through all stages of oogenesis. Active Caspase-3 results show that the amount of germ cell apoptosis is lower in double homozygous *fancl^−/−^;tp53^−/−^* individuals than in their *fancl* ^−/−^;*tp53* ^+/+^ mutant sibling controls, which further supports the hypothesis that the abnormal increase of apoptosis in *fancl* mutants that compromises the survival of meiotic oocytes is the mechanism responsible for the female-to-male sex reversal.

We did not notice a sex ratio biased towards females in the *tp53* ***^M214K^*** mutant line. This allele, however, is hypomorphic, and may possess levels of apoptosis compatible with the male pathway. This conclusion is supported by our finding that a few *fancl^−/−^;tp53^−/−^* double mutants developed as males. An alternative explanation is that mechanisms of apoptosis independent of Tp53 might occur in male gonads that promote oocytes to disappear in developing testes.

### Mutation of Fanconi Anemia Genes Promotes Activation of Tp53-Mediated Apoptosis in Both Zebrafish and Mice

Our finding of increased germ cell apoptosis in *fancl* zebrafish mutants is consistent with the increase of apoptosis in a variety of cell types reported in Fanconi Anemia knockout mice. For instance, *Fanca* ^−/−^, *Fancc* ^−/−^, and *Fancg* ^−/−^ knockout mice show increased apoptosis of hematopoietic or neuronal cells, which might lead to a progressive loss of stem and progenitor cells [68]–[70]. Bone marrow failure in children with Fanconi Anemia is attributed to excessive apoptosis and subsequent failure of the hematopoietic stem cell compartment (reviewed in [56]). Interestingly, *Fanca^−/−^* knockout mice also show increased male germ cell apoptosis [37], suggesting that a role of the FA network related to apoptosis of germ cells might be a conserved feature in fish and mammals. Young *Fancl* ^−/−^ knockout mice, in contrast to *fancl* mutant zebrafish, do not show sex reversal but initially develop as sterile males and sterile females. *Fancl* ^−/−^ knockout male mice – but significantly, not *Fancl* ^−/−^ knockout female mice – can recover fertility and become fertile adult males. These results suggest that Fancl is necessary for germ cell proliferation in mouse embryos and for the maturation of oocytes, but not for the proliferation or maturation of spermatogonia in adulthood [58]. In zebrafish, the fact that *fancl* mutant males are fertile and that *fancl^−/−^;tp53^−/−^* rescued females are also fertile indicates that Fancl function is not essential for the maturation of zebrafish spermatogonia and oogonia to become sperm or mature oocytes, but rather that Fancl function affects specifically germ cell survival.

The loss of oocytes progressing through meiosis in *fancl* mutants suggests that Fancl function is involved in the survival of developing germ cells through meiosis, and that when Fancl is mutated, developing oocytes cannot survive due to an inappropriate increase of Tp53-dependent germ cell apoptosis. This idea is consistent with the fact that genetic deletion of Tp53 can rescue the TNF-alpha dependent apoptosis caused by accumulation of the pro-apoptotic protein kinase PKR resulting from a mutation of the human *FANCC* gene [68], reviewed in [56]. Therefore, inappropriate activation of Tp53-dependent apoptosis might be a common mechanism affecting cell survival in both zebrafish and human after alteration of the FA network. Given the fundamental similarity of the cellular mechanisms of the FA pathway in zebrafish and humans, the screening of small molecule libraries for compounds that can rescue the sex-reversal phenotype of zebrafish *fancl* mutants might identify compounds of therapeutic importance for Fanconi Anemia patients.

### A Model for Zebrafish Sex Determination: Oocyte Survival Regulated by Tp53-Mediated Apoptosis Can Alter Gonad Fate

**Figure 8:**
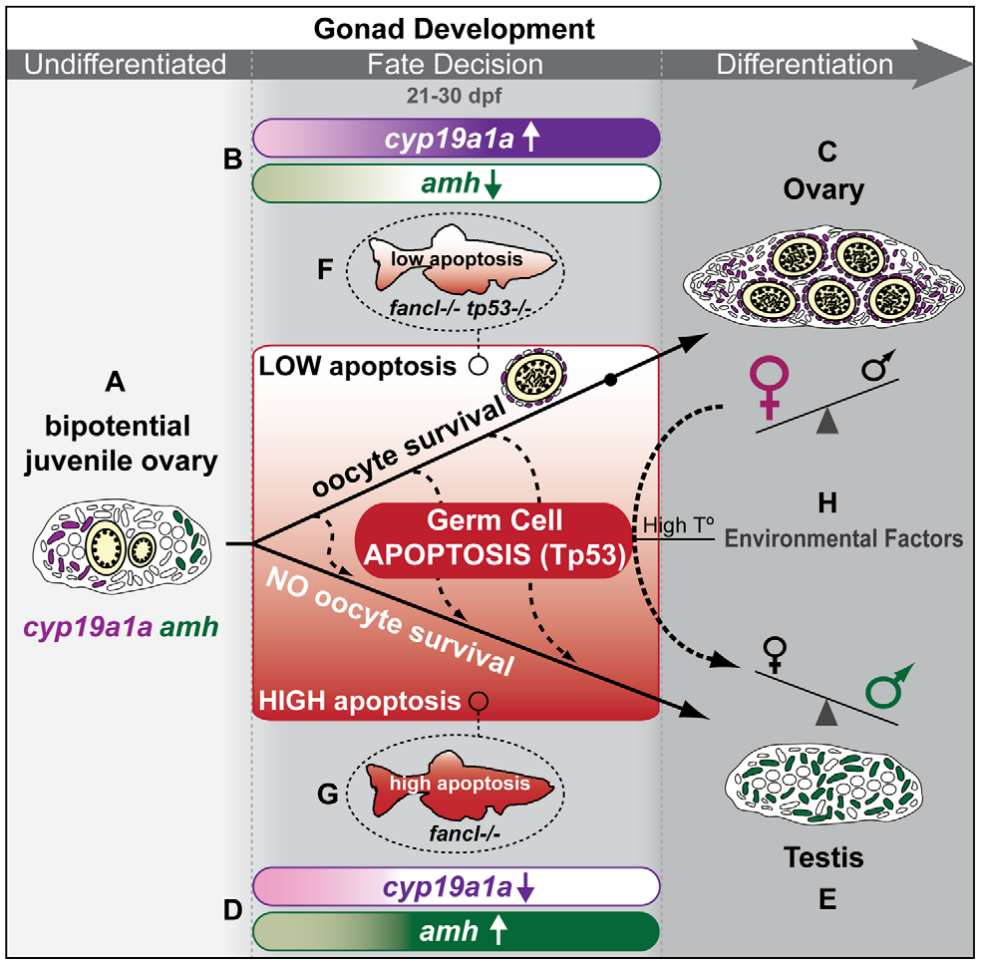
A model for zebrafish sex determination: oocyte survival regulated by Tp53-mediated apoptosis can alter gonad fate. This model suggests germ cell apoptosis as a central feature that can integrate genetic and environmental factors to tip the fate of the gonad towards the female or the male pathway and thus determine zebrafish sex. (A) Zebrafish juveniles initially develop an undifferentiated bipotential immature ovary regardless of their eventual definitive sex. The juvenile gonad contains developing oocytes (shown in yellow), as well as somatic cells that express female-specific markers like *cyp19a1a* (purple) and early male-specific markers like *amh* (green). This model suggests that different levels of germ cell apoptosis (indicated as a red gradient box from low (white) to high apoptosis (red)) has the potential to tip the fate of the gonad: high apoptosis (e.g *fancl^−/−^* mutants) tips fate towards the male pathway, while low apoptosis (e.g. *fancl^−/−^ tp53^−/−^* mutants) tips fate towards the female pathway and rescues the sex-reversal phenotype of *fancl* mutants. In this model, wild-type zebrafish can enter the male pathway at different times during the fate decision time-window (dashed arrows in apoptosis box) ([30] and this work), which is probably related to the level of apoptosis that affects oocyte survival in each particular individual. (B) Analysis of somatic markers reported here shows that the survival of oocytes during the fate decision time-window is crucial to maintain and increase expression of *cyp19a1a* in the somatic cells of the gonad (B, purple gradient) and to down-regulate the expression of *amh* in the somatic cells of the gonad (B, green gradient), perhaps due to an oocyte-derived signal, which in *fancl* mutants would be compromised. (C) This gene expression profile feminizes the gonad, oocytes continue to develop, the gonad differentiates as an ovary, and the individual becomes a female. (D) In the absence of oocytes during sex fate decision time, as in *fancl* mutants, gonads do not maintain *cyp19a1a* (D, purple gradient), but instead up-regulate *amh* expression (D, green gradient). (E) This gene expression profile masculinizes the gonad, which differentiates as a mature testis and the individual becomes a male. (F,G) The absence of surviving oocytes in *fancl* ^−/−^ mutants is probably due to high levels of germ cell apoptosis, which causes all animals to develop as males due to female-to-male sex reversal. This sex reversal phenotype can be rescued by decreasing germ cell apoptosis in double homozygous *fancl* ^−/−^;*tp53* ^−/−^ mutants. Therefore, our analysis of *fancl* mutants provides evidence supporting the model that the survival of developing oocytes through meiosis, and not the mere presence of germ cells, is a critical factor that tips the fate of the gonad towards the female pathway in zebrafish. (H) Other work has shown that environmental factors such as high temperature can also induce oocyte apoptosis and tip the fate of the gonads towards the male pathway [71]. In light of this analysis, our model suggests that sex-determining mechanisms in zebrafish integrate signals from genetic and environmental factors that can modify the levels of Tp53-mediated germ cell apoptosis, which influence oocyte survival during the period of gonad fate decision, and tip the fate of the gonad towards the female or the male pathway, thus determining the sex of zebrafish.

Our analysis of zebrafish *fancl* mutants suggests a model in which oocyte survival regulated by Tp53-mediated apoptosis is a central element that can tip gonad fate towards the male or the female pathway (gradient red box in Figure 8). Zebrafish develop initially as juvenile hermaphrodites, and have immature ovaries during the juvenile stage regardless of their definitive sex [26]–[28]. This immature ovary is bipotential, and expresses both female (*cyp19a1a*) and male (*amh*) specific markers (Figure 8A) [29], [31], [48]. During the fate decision period, some wild-type animals up-regulate *cyp19a1a* and suppress *amh* expression (Figure 8B) thereby tipping the fate of the gonad towards the female ovarian pathway (Figure 8C). Complementarily, other wild-type individuals suppress *cyp19a1a* and up-regulate *amh* expression (Figure 8D) and gonad fate tips towards the male testis pathway (Figure 8E). In this work, we show that oocyte survival is crucial to maintain the female gene expression profile of somatic cells that is essential for ovary development.

In wild-type zebrafish, juvenile bipotential gonads contain immature oocytes at early stage IB ([49]; and this work). In transitional stages, gonads that become ovaries possess oocytes that progress through meiosis to late stage IB and reach diplotene, where they arrest for the remainder of oocyte development [49]. In *fancl^−/−^* homozygous mutants, loss of oocytes at or before diplotene likely alters signaling from germ line to the soma, leading to loss of *cyp19a1a* expression, failure to down regulate *amh* expression, and consequent masculinization of the gonads to form testes (Figure 8G). The *cyp19a1a* gene encodes aromatase, the enzyme that converts testosterone to estrogen. It is known that aromatase is critical for female fate in zebrafish because pharmacological treatments with the aromatase inhibitor fadrozole masculinizes gonads [71]–[73] and because, complementarily, treatments with estrogen (estradiol) down-regulate *amh* expression and feminize the gonad [74]. We hypothesize that the apoptotic loss of oocytes in *fancl* mutants causes *cyp19a1a* gene expression to disappear and leads to the failure to maintain aromatase levels, which results in failure to produce and sustain high estrogen levels in the gonad, causing gonads to abandon the female fate and instead, enter the testis developmental program.

The presence of oocytes appears to be important for sex determination not only for zebrafish, but also for medaka. In contrast to zebrafish, in which all individuals begin oogenesis, in medaka only XX females start oogenesis while XY males suppress oogenesis and all germ cells remain undifferentiated (reviewed in [75]). A feature common to both species is that the number of developing oocytes is a key feature that tips undifferentiated gonads towards an ovary fate ([31], [75] and this work). In medaka, the partial removal of PGCs can reduce the number of developing oocytes below a threshold necessary for female development [76]. In addition, medaka *hotei* mutants, which have aberrant oocyte development [77], fail to maintain *cyp19a1a* expression and gonads develop into testes. Therefore, the survival of developing oocytes appears to be important for sex determination in both zebrafish and medaka. These considerations support the hypothesis that when the number of oocytes exceeds a threshold, sexual fate tips towards the female pathway, and alternatively, when the oocyte number fails to exceed that threshold, the sexual fate tips towards the male pathway, as we observed in zebrafish *fancl* mutants.

In zebrafish, presumptive juvenile males had more TUNEL signal in germ cells than presumptive females had suggesting the hypothesis that oocyte apoptosis could be the mechanism of testicular and ovarian differentiation in zebrafish [27]. Consistent with this hypothesis, analysis of *ziwi* null mutants showed that total loss of germ cells by apoptosis caused *ziwi* mutants to develop exclusively as sterile males [34]. Our results show that Tp53-mediated germ cell apoptosis is a mechanism that can tip gonad fate towards the female or male pathway, at least in *fancl* mutants. Because environmental factors such as high temperature (Figure 8H) or endocrine-disrupting chemical treatments can also increase oocyte apoptosis and cause sex reversal [71]–[73], it is plausible to suggest that the integration of genetic and environmental factors converge to modify the levels of Tp53-mediated germ cell apoptosis, which affect oocyte survival during the critical time window to determine the sexual fate of the gonad, and ultimately alter zebrafish sex determination.

## Materials and Methods

### Ethics Statement

Animals were handled in accordance with good animal practice as defined by relevant animal welfare bodies, and the University of Oregon Institutional Animal Care and Use Committee approved all animal work (Animal Welfare Assurance Number A-3009-01, IACUC protocol #08-13).

### Animals

The zebrafish *fancl* mutation (*HG10A*; GenBank accession AB353980) was generated by insertional mutagenesis by Tol2 transposon-mediated enhancer trap [42]. The *tp53* mutant line *tp53^zdf1^* causing the amino acid substitution M214K was obtained from ZIRC (http://zebrafish.org/zirc/home/guide.php) [55]. Genotyping of *tp53* animals was performed as described [55]. Genetic nomenclature follows guidelines from ZFIN (http://zfin.org/zf_info/nomen.html).

### Genotyping of *fancl* Mutants

The full-length zebrafish *fancl* cDNA was previously described [44] (GenBank accession AY968598). Primer pairs used to amplify the *fancl* wild-type or mutant alleles were: WT_F:CTGGTCTTTATTGACTGTAATGGC; WT_R:TAGATAAGCTCCAGATTTGGCTTG; Mutant_F:GTCAGCCCATCCAGATCAGCAG; Mutant_R:CATGACGTCACTTCCAAAGGACC. PCR conditions were: 5′94°C; 32 cycles of: 30″94°C, 30″55°C, 1′72°C; followed by 10′72°C. Sizes of PCR-amplified bands: Wild type: 479 bp; Mutant: 370 bp.

### Reverse Transcriptase–PCR

Total RNA isolation from dissected adult testes and cDNA synthesis were performed as described [41]. Primers used for reverse transcriptase-PCR (RT-PCR) experiments were: F1:GACGGCTTCATCACAGTGCTG; R1:CATGACGTCACTTCCAAAGGACC; F2:GAACCCTGACTGCACTGTCCTAC; R2:GCTTTGGCGACTGGTTGGCAGAC. PCR conditions were: F1-R1: 3′94°C; 40 cycles of: 30″94°C, 30″58°C, 1′30″72°C; followed by 10′72°C; F2-R2: 3′94°C; 37 cycles of: 20″94°C, 30″60°C, 45″72°C; followed by 10′72°C. Sizes of PCR-amplified bands: F1-R1: 1239 bp F2-R2: 232 bp.

### *dead end* Morpholino Injections

To obtain animals lacking germ cells, wild-type zebrafish embryos from the AB strain were injected at the 1–2 cell stage with antisense morpholino oligonucleotide (Gene Tools, Oregon) directed against *dead end* as described [46]. Sibling non-injected embryos and a fraction of *dnd* MO-injected embryos were fixed at 24 hours post-fertilization to confirm the presence or absence of germ cells by whole-mount in situ hybridization using *vasa* probe as described [47].

### In Situ Hybridization and Histology

Animals were reared and collected under standard conditions [78]. In situ hybridization experiments on zebrafish cryosections were performed as described [29]. Adjacent sections of gonads were obtained by placing three consecutive sections of the gonad on three different slides. Probes for *amh and cyp19a1a* were made as described [29] and probe for *vasa* was made from its 3′end as described [47]. A *fancl* cDNA fragment of 786 nt containing the PHD domain (nucleotides 646-1431 of AY968598) was cloned in TOPO vector (Invitrogen) and used to synthesize DIG-labeled riboprobe (Boehringer Mannheim). For gonad histology, euthanized animals were fixed in Bouin's fixative for about 24–48 hours and washed repeatedly in 70% ethanol. Animals were dehydrated and embedded in paraffin, sectioned at 7 microns, and stained with hematoxylin and eosin.

### Immunohistochemistry

Animals were fixed at 25 dpf in 4% PFA ON at 4°C, dehydrated, embedded in paraffin, and sectioned at 7 microns. Apoptotic cells were detected by immuno-fluorescence using anti-active Caspase-3 as primary antibody (1∶200, BD Pharmingen) and Alexa-Fluor594 goat anti-rabbit as secondary antibody (1∶1000, Invitrogen) following an immuno-histochemical protocol (S. Cheesman, personal communication). Gonads were screened for positive signal by DIC-fluorescence microscopy. The number of positive cells in gonads of *fancl* and wild-type animals was scored in 840 sections: 70 sections containing gonads per each animal (n = 12).

## References

[1] Marshall Graves JA (2008). Weird animal genomes and the evolution of vertebrate sex and sex chromosomes.. Annu Rev Genet.

[2] Devlin RH, Nagahama Y (2002). Sex determination and sex differentiation in fish: an overview of genetic, physiological, and environmental influences.. Aquaculture.

[3] Volff JN, Schartl M (2001). Variability of genetic sex determination in poeciliid fishes.. Genetica.

[4] Ross JA, Urton JR, Boland J, Shapiro MD, Peichel CL (2009). Turnover of sex chromosomes in the stickleback fishes (gasterosteidae).. PLoS Genet.

[5] Kitano J, Ross JA, Mori S, Kume M, Jones FC (2009). A role for a neo-sex chromosome in stickleback speciation.. Nature.

[6] Ser JR, Roberts RB, Kocher TD (2009). Multiple Interacting Loci Control Sex Determination in Lake Malawi Cichlid Fish.. Evolution.

[7] Davidson WS, Huang TK, Fujiki K, von Schalburg KR, Koop BF (2009). The sex determining loci and sex chromosomes in the family salmonidae.. Sex Dev.

[8] Barske LA, Capel B (2008). Blurring the edges in vertebrate sex determination.. Curr Opin Genet Dev.

[9] Baroiller JF, D'Cotta H, Saillant E (2009). Environmental effects on fish sex determination and differentiation.. Sex Dev.

[10] Sato T, Endo T, Yamahira K, Hamaguchi S, Sakaizumi M (2005). Induction of female-to-male sex reversal by high temperature treatment in Medaka, Oryzias latipes.. Zoolog Sci.

[11] Marin I, Baker BS (1998). The evolutionary dynamics of sex determination.. Science.

[12] Gubbay J, Collignon J, Koopman P, Capel B, Economou A (1990). A gene mapping to the sex-determining region of the mouse Y chromosome is a member of a novel family of embryonically expressed genes.. Nature.

[13] Sinclair AH, Berta P, Palmer MS, Hawkins JR, Griffiths BL (1990). A gene from the human sex-determining region encodes a protein with homology to a conserved DNA-binding motif.. Nature.

[14] Koopman P, Gubbay J, Vivian N, Goodfellow P, Lovell-Badge R (1991). Male development of chromosomally female mice transgenic for Sry.. Nature.

[15] Sekido R, Lovell-Badge R (2009). Sex determination and SRY: down to a wink and a nudge?. Trends Genet.

[16] Williams TM, Carroll SB (2009). Genetic and molecular insights into the development and evolution of sexual dimorphism.. Nat Rev Genet.

[17] Brennan J, Capel B (2004). One tissue, two fates: molecular genetic events that underlie testis versus ovary development.. Nat Rev Genet.

[18] Wallis MC, Waters PD, Delbridge ML, Kirby PJ, Pask AJ (2007). Sex determination in platypus and echidna: autosomal location of SOX3 confirms the absence of SRY from monotremes.. Chromosome Res.

[19] Matsuda M, Nagahama Y, Shinomiya A, Sato T, Matsuda C (2002). DMY is a Y-specific DM-domain gene required for male development in the medaka fish.. Nature.

[20] Nanda I, Kondo M, Hornung U, Asakawa S, Winkler C (2002). A duplicated copy of DMRT1 in the sex-determining region of the Y chromosome of the medaka, Oryzias latipes.. Proc Natl Acad Sci U S A.

[21] Smith CA, Roeszler KN, Ohnesorg T, Cummins DM, Farlie PG (2009). The avian Z-linked gene DMRT1 is required for male sex determination in the chicken.. Nature.

[22] Kondo M, Nanda I, Hornung U, Asakawa S, Shimizu N (2003). Absence of the candidate male sex-determining gene dmrt1b(Y) of medaka from other fish species.. Curr Biol.

[23] Schreeb KH, Broth G, Sachsse W, Freundt KJ (1993). The karyotype of the zebrafish (*Brachydanio rerio*).. J Exp Anim Sci.

[24] Pijnacker LP, Ferwerda MA (1995). Zebrafish chromosome banding.. Genome.

[25] Amores A, Postlethwait JH, Detrich HW, Westerfield M, Zon LI (1999). Banded chromosomes and the zebrafish karyotype.. The Zebrafish: Genetics and Genomics.

[26] Takahashi H (1977). Juvenile hermaphroditism in the zebrafish, Brachydanio rerio.. Bull Fac Fish Hokkaido Univ.

[27] Uchida D, Yamashita M, Kitano T, Iguchi T (2002). Oocyte apoptosis during the transition from ovary-like tissue to testes during sex differentiation of juvenile zebrafish.. J Exp Biol.

[28] Maack G, Segner H (2003). Morphological development of the gonads in zebrafish.. Journal of Fish Biology.

[29] Rodriguez-Mari A, Yan YL, Bremiller RA, Wilson C, Canestro C (2005). Characterization and expression pattern of zebrafish Anti-Mullerian hormone (Amh) relative to sox9a, sox9b, and cyp19a1a, during gonad development.. Gene Expr Patterns.

[30] Wang XG, Bartfai R, Sleptsova-Freidrich I, Orban L (2007). The timing and extent of ‘juvenile ovary’ phase are highly variable during zebrafish testis differentiation.. Journal of Fish Biology.

[31] Siegfried KR, Nusslein-Volhard C (2008). Germ line control of female sex determination in zebrafish.. Dev Biol.

[32] Slanchev K, Stebler J, de la Cueva-Mendez G, Raz E (2005). Development without germ cells: the role of the germ line in zebrafish sex differentiation.. Proc Natl Acad Sci U S A.

[33] Draper BW, McCallum CM, Moens CB (2007). nanos1 is required to maintain oocyte production in adult zebrafish.. Dev Biol.

[34] Houwing S, Kamminga LM, Berezikov E, Cronembold D, Girard A (2007). A role for Piwi and piRNAs in germ cell maintenance and transposon silencing in Zebrafish.. Cell.

[35] Moldovan GL, D'Andrea AD (2009). How the fanconi anemia pathway guards the genome.. Annu Rev Genet.

[36] Zhang X, Li J, Sejas DP, Rathbun KR, Bagby GC (2004). The Fanconi anemia proteins functionally interact with the protein kinase regulated by RNA (PKR).. J Biol Chem.

[37] Wong JC, Alon N, McKerlie C, Huang JR, Meyn MS (2003). Targeted disruption of exons 1 to 6 of the Fanconi Anemia group A gene leads to growth retardation, strain-specific microphthalmia, meiotic defects and primordial germ cell hypoplasia.. Hum Mol Genet.

[38] Auerbach AD (2009). Fanconi anemia and its diagnosis.. Mutat Res.

[39] Meetei AR, de Winter JP, Medhurst AL, Wallisch M, Waisfisz Q (2003). A novel ubiquitin ligase is deficient in Fanconi anemia.. Nat Genet.

[40] Gurtan AM, Stuckert P, D'Andrea AD (2006). The WD40 repeats of FANCL are required for Fanconi anemia core complex assembly.. J Biol Chem.

[41] Titus TA, Yan YL, Wilson C, Starks AM, Frohnmayer JD (2009). The Fanconi anemia/BRCA gene network in zebrafish: embryonic expression and comparative genomics.. Mutat Res.

[42] Nagayoshi S, Hayashi E, Abe G, Osato N, Asakawa K (2008). Insertional mutagenesis by the Tol2 transposon-mediated enhancer trap approach generated mutations in two developmental genes: tcf7 and synembryn-like.. Development.

[43] Coscoy L, Ganem D (2003). PHD domains and E3 ubiquitin ligases: viruses make the connection.. Trends Cell Biol.

[44] Titus TA, Selvig DR, Qin B, Wilson C, Starks AM (2006). The Fanconi anemia gene network is conserved from zebrafish to human.. Gene.

[45] Ciruna B, Weidinger G, Knaut H, Thisse B, Thisse C (2002). Production of maternal-zygotic mutant zebrafish by germ-line replacement.. Proc Natl Acad Sci U S A.

[46] Weidinger G, Stebler J, Slanchev K, Dumstrei K, Wise C (2003). dead end, a novel vertebrate germ plasm component, is required for zebrafish primordial germ cell migration and survival.. Curr Biol.

[47] Yoon C, Kawakami K, Hopkins N (1997). Zebrafish vasa homologue RNA is localized to the cleavage planes of 2- and 4-cell-stage embryos and is expressed in the primordial germ cells.. Development.

[48] Wang XG, Orban L (2007). Anti-Mullerian hormone and 11 beta-hydroxylase show reciprocal expression to that of aromatase in the transforming gonad of zebrafish males.. Dev Dyn.

[49] Selman K, Wallace RA, Sarka A, Qi X (1993). Stages of oocyte development in the zebrafish, *Brachydanio rerio*.. Journal of Morphology.

[50] Baumeister HG (1973). Lampbrush chromosomes and RNA-synthesis during early oogenesis of Brachydanio rerio (Cyprinidae, Teleostei).. Z Zellforsch Mikrosk Anat.

[51] Mc Millan DB (2007). Fish Histology: Female Reproductive Systems..

[52] Dai C, Krantz SB (1999). Interferon gamma induces upregulation and activation of caspases 1, 3, and 8 to produce apoptosis in human erythroid progenitor cells.. Blood.

[53] Ryu S, Holzschuh J, Erhardt S, Ettl AK, Driever W (2005). Depletion of minichromosome maintenance protein 5 in the zebrafish retina causes cell-cycle defect and apoptosis.. Proc Natl Acad Sci U S A.

[54] Fridman JS, Lowe SW (2003). Control of apoptosis by p53.. Oncogene.

[55] Berghmans S, Murphey RD, Wienholds E, Neuberg D, Kutok JL (2005). tp53 mutant zebrafish develop malignant peripheral nerve sheath tumors.. Proc Natl Acad Sci U S A.

[56] Parmar K, D'Andrea A, Niedernhofer LJ (2009). Mouse models of Fanconi anemia.. Mutat Res.

[57] Agoulnik AI, Lu B, Zhu Q, Truong C, Ty MT (2002). A novel gene, Pog, is necessary for primordial germ cell proliferation in the mouse and underlies the germ cell deficient mutation, gcd.. Hum Mol Genet.

[58] Lu B, Bishop CE (2003). Late onset of spermatogenesis and gain of fertility in POG-deficient mice indicate that POG is not necessary for the proliferation of spermatogonia.. Biol Reprod.

[59] Houwing S, Berezikov E, Ketting RF (2008). Zili is required for germ cell differentiation and meiosis in zebrafish.. Embo J.

[60] Baron D, Houlgatte R, Fostier A, Guiguen Y (2005). Large-scale temporal gene expression profiling during gonadal differentiation and early gametogenesis in rainbow trout.. Biol Reprod.

[61] Yang YG, Herceg Z, Nakanishi K, Demuth I, Piccoli C (2005). The Fanconi anemia group A protein modulates homologous repair of DNA double-strand breaks in mammalian cells.. Carcinogenesis.

[62] Yao HH, DiNapoli L, Capel B (2003). Meiotic germ cells antagonize mesonephric cell migration and testis cord formation in mouse gonads.. Development.

[63] McLaren A, Southee D (1997). Entry of mouse embryonic germ cells into meiosis.. Dev Biol.

[64] McLaren A (1991). Development of the mammalian gonad: the fate of the supporting cell lineage.. Bioessays.

[65] Albrecht KH, Eicher EM (2001). Evidence that Sry is expressed in pre-Sertoli cells and Sertoli and granulosa cells have a common precursor.. Dev Biol.

[66] Nakamura S, Aoki Y, Saito D, Kuroki Y, Fujiyama A (2008). Sox9b/sox9a2-EGFP transgenic medaka reveals the morphological reorganization of the gonads and a common precursor of both the female and male supporting cells.. Mol Reprod Dev.

[67] Guigon CJ, Magre S (2006). Contribution of germ cells to the differentiation and maturation of the ovary: insights from models of germ cell depletion.. Biol Reprod.

[68] Freie B, Li X, Ciccone SL, Nawa K, Cooper S (2003). Fanconi anemia type C and p53 cooperate in apoptosis and tumorigenesis.. Blood.

[69] Rio P, Segovia JC, Hanenberg H, Casado JA, Martinez J (2002). In vitro phenotypic correction of hematopoietic progenitors from Fanconi anemia group A knockout mice.. Blood.

[70] Sii-Felice K, Etienne O, Hoffschir F, Mathieu C, Riou L (2008). Fanconi DNA repair pathway is required for survival and long-term maintenance of neural progenitors.. Embo J.

[71] Uchida D, Yamashita M, Kitano T, Iguchi T (2004). An aromatase inhibitor or high water temperature induce oocyte apoptosis and depletion of P450 aromatase activity in the gonads of genetic female zebrafish during sex-reversal.. Comp Biochem Physiol A Mol Integr Physiol.

[72] Fenske M, Segner H (2004). Aromatase modulation alters gonadal differentiation in developing zebrafish (Danio rerio).. Aquat Toxicol.

[73] Villeneuve L, Wang RL, Bencic DC, Biales AD, Martinovic D (2009). Altered gene expression in the brain and ovaries of zebrafish (Danio rerio) exposed to the aromatase inhibitor fadrozole: microarray analysis and hypothesis generation.. Environ Toxicol Chem.

[74] Schulz RW, Bogerd J, Male R, Ball J, Fenske M (2007). Estrogen-induced alterations in amh and dmrt1 expression signal for disruption in male sexual development in the zebrafish.. Environ Sci Technol.

[75] Saito D, Tanaka M (2009). Comparative aspects of gonadal sex differentiation in medaka: a conserved role of developing oocytes in sexual canalization.. Sex Dev.

[76] Kurokawa H, Saito D, Nakamura S, Katoh-Fukui Y, Ohta K (2007). Germ cells are essential for sexual dimorphism in the medaka gonad.. Proc Natl Acad Sci U S A.

[77] Morinaga C, Saito D, Nakamura S, Sasaki T, Asakawa S (2007). The hotei mutation of medaka in the anti-Mullerian hormone receptor causes the dysregulation of germ cell and sexual development.. Proc Natl Acad Sci U S A.

[78] Westerfield M (1995). The zebrafish book: a guide for the laboratory use of zebrafish (*Danio rerio*)..

